# A Mode-Localized Micro-Electromechanical System Accelerometer with Force Rebalance Closed-Loop Control

**DOI:** 10.3390/mi16030248

**Published:** 2025-02-21

**Authors:** Bowen Wang, Zhenxiang Qi, Kunfeng Wang, Zhaoyang Zhai, Zheng Wang, Xudong Zou

**Affiliations:** 1 The State Key Laboratory of Transducer Technology, Aerospace Information Research Institute, Chinese Academy of Sciences, Beijing 100190, China; wangbowen20@mails.ucas.ac.cn (B.W.); qizhenxiang21@mails.ucas.ac.cn (Z.Q.); wangkunfeng17@mails.ucas.ac.cn (K.W.); zhaizhaoyang20@mails.ucas.ac.cn (Z.Z.); 2 School of Electronic, Electrical and Communication Engineering, University of Chinese Academy of Sciences, Beijing 100049, China; 3 QiLu Aerospace Information Research Institute, Jinan 250132, China; wangzheng02@aircas.ac.cn

**Keywords:** MEMS accelerometer, mode localization, amplitude ratio, force rebalance

## Abstract

This article proposes a force rebalance control scheme based on a mode-localized resonant accelerometer (ML-RXL), which is applied to address the limited measurement range problem of the ML-RXL. For the first time, an empirical response model of the weakly coupling resonators for the amplitude ratio output is established. Based on this, this paper builds an overall model of the force rebalance control system to analyze the sensitivity characteristics by simulations, which demonstrates that the scheme can effectively broaden the linear measurement range. It is demonstrated that the sensor exhibits a highly linear output within a measurement range of ±1 g, with a sensitivity of the feedback-control voltage output measured at 2.94 V/g. The measurement range is expanded by at least 6.7 times. Moreover, the results show that the minimum input-referred acceleration noise density of the sensor for the force rebalance control scheme is 3.29 μg/rtHz, and that the best bias instability is optimized to 5.34 μg with an integral time of 0.64 s.

## 1. Introduction

Conventional acceleration sensors primarily include fiber-optic accelerometers [1,2], capacitive accelerometers [3,4,5], and resonant accelerometers [6,7]. Fiber-optic accelerometers maintain stable measurement performance in environments with strong electromagnetic interference; however, the manufacturing and integration costs of fiber-optics are high, and the process is complex. Capacitive accelerometers are stable with low temperature drift but require a high detection capability for small capacitance variation. Resonant accelerometers output frequency-modulated signals, which are easily digitized, providing good resolution. Furthermore, accelerometers based on the mode-localization effect have become a focal point for researchers’ attention in recent years to further enhance detection sensitivity [8,9,10,11,12]. Mode-localization is a phenomenon of energy transfer occurring in periodic systems, which has been studied since the 1950s [13,14]. In a periodic system, if there is a small disturbance in the original equilibrium system, the propagation of vibrations will be confined to a small region in space, known as mode-localization. This phenomenon is now widely utilized in the development of other high-sensitivity micro-electromechanical system (MEMS) sensors, including mass sensors [15,16], electric-field sensors [17], and magnetic sensors [18], among other sensors.

For the mode-localized resonant accelerometer (ML-RXL), the core structure consists of weakly coupling resonators (WCRs). When WCRs operate, the energy is confined within the resonators and distributed among different resonators. When the WCRs are subjected to the stiffness perturbation, the energy is redistributed between the resonators through the coupling structure, which can result in significant changes to intrinsic-mode displacements. Therefore, the changes in the amplitude ratio (AR) for the WCRs can be monitored. Research on the ML-RXL has become increasingly comprehensive, and researchers employ various methods to significantly enhance the parameter sensitivity of the ML-RXL, including structure optimization [19,20,21,22,23,24,25], electrostatic tuning [11,26,27], nonlinear effects [28,29], internal resonance modulation [30], and pump modulation [31]. Additionally, the common-mode suppression capability of the ML-RXL against environmental drift (temperature, pressure) has been validated [32,33,34,35,36]. Moreover, an ML-RXL designed specifically for *z*-axis acceleration detection has been reported [37], indicating its great potential for realizing chip-integrated triaxial accelerometers.

However, the AR output exhibits significant nonlinear characteristics near the bifurcation point owing to its intrinsic nature [35,38]. Moreover, when the AR is relatively large, the constraints of the signal-to-noise ratio (SNR) can also lead to the presence of nonlinear relationships, resulting in a narrow effective measurement range [21,39]. Chang et al. have proposed using the amplitude difference (AD) as an output metric to achieve linear sensing across the bifurcation point [40]. However, this approach, similarly to the AR output, has a limited linear sensing range. In addition, using the algebraic summation of amplitude ratios as the output matrix can also extend the measurement range [41,42]. Currently, the implementation of this method necessitates the additional WCRs. Guo et al. introduced a novel output metric that is derived from the subtraction of reciprocal amplitude ratios (SRARs) [43,44]. This approach aims to achieve theoretically full-scale linear sensing for an ML-RXL, but its measurement range is still affected by the nonlinearity constrained of the SNR.

The force rebalance control scheme is widely used in capacitive accelerometers [3,4,5]. In the open-loop state, the sensitivity of the capacitive accelerometer is contradictory to the measurement range, bandwidth, and linearity. By working in the force rebalance closed-loop state, the capacitive accelerometer can not only improve the linearity and range of the system, but meet the requirements of the bandwidth and the dynamic characteristics of the system.

In this innovative work, a force rebalance control strategy for an ML-RXL was first proposed to address the challenge of limited measurement range inherent to this type of device. This strategy utilizes a feedback-control voltage signal as the output, which accurately represents the inertial acceleration experienced by the device. Furthermore, it effectively counteracts the inertial forces by applying electrostatic forces to the proof mass, thereby ensuring that the amplitude ratio is maintained at a predetermined stable value. In comparison to several previously discussed methods designed to enhance the measurement range, this novel approach effectively decouples the sensitivity and measurement range of the WCRs. This strategy not only adeptly circumvents the negative impacts associated with theoretical nonlinear bifurcation zones but mitigates nonlinear phenomena in the amplitude ratio output when subjected to large input signals. Consequently, while preserving its ultra-high sensitivity detection capabilities, this solution significantly broadens the measurement range.

Following the introduction, this paper elaborates the working principle of an ML-RXL based on the force rebalance scheme and conducts a theoretical stability analysis of this control system in the Section 2. In the Section 3, a complete model of the force rebalance system is established in simulation, and the control parameters of the system are then determined based on the theoretical analysis, so as to analyze the sensitivity characteristics of the device. Section 4 describes the device fabrication and the experimental setup. Finally, the characterizations of the scale factor, the bias-stability, and the noise floor are shown, as measured by the sensor, in the Section 5.

## 2. Theory Analysis

### 2.1. Working Principle

**Figure 1:**
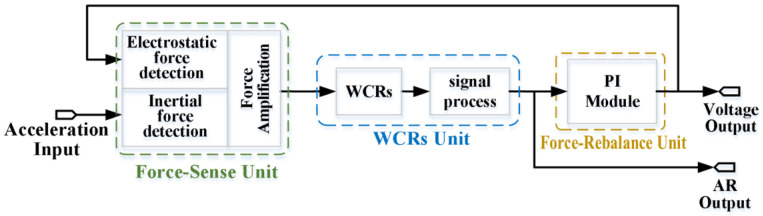
The schematic diagram of the force rebalance scheme for the ML-RXL.

The concrete working principle of the ML-RXL based on the force rebalance control scheme is designed, as shown in Figure 1. The sensor mainly comprises structures for inertial force detection and electrostatic force detection. When the force detection structure is subjected to an acceleration along the sensitive axis direction, the inertial force is amplified and subsequently applied to the WCRs. And thus, the stiffnesses of the resonators are changed, and it leads to the mode-localization effect, which results in the AR shift of the WCRs.

When the ML-RXL operates under the force rebalance control scheme, the feedback-control voltage is applied to the specific feedback electrodes to generate electrostatic force acting on the detection structure. The electrostatic force aims to counteract the inertial force, thereby maintaining the AR of the WCRs near a constant value. Therefore, the feedback-control voltage, serving as the output of the ML-RXL, reflects the magnitude of the inertial acceleration.

### 2.2. 2-DoF WCRs

**Figure 2:**
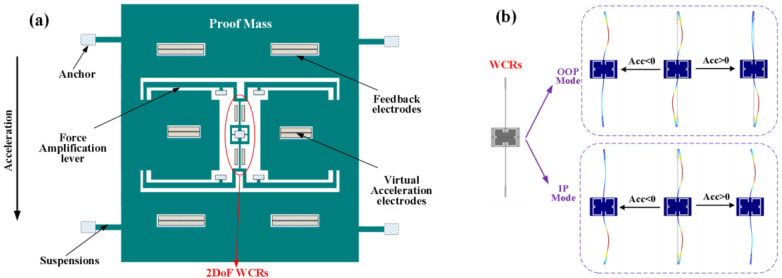
(**a**) Schematic diagram of the ML-RXL, (**b**) Mode shapes of 2DoF for WCRs under different perturbation conditions.

This structure of the ML-RXL mainly comprises the movable proof mass, suspensions, the force-amplification level, 2DoF for WCRs, virtual acceleration electrodes, and feedback electrodes, as shown in Figure 2a. The WCRs achieve a weak coupling stiffness based on a mechanical method. Therefore, there are two coupled modes with similar frequencies: an in-phase (IP) mode and an out-of-phase (OOP) mode.

**Figure 3:**
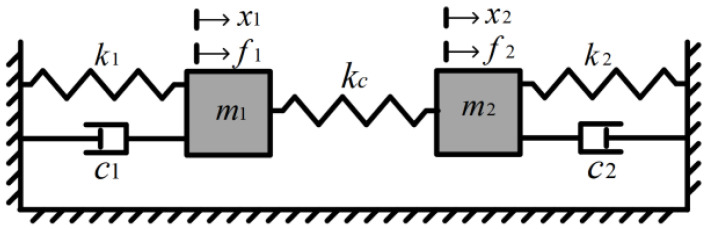
Mass-spring-damper model of the 2DoF for WCRs.

The 2DoF for WCRs are simplified as a mass-spring-damper system (Figure 3), for which the dynamic equations of the model can be described as follows:

(1) $$\left\{\begin{array}{c} m_{1}{\ddot{x}}_{1}+c_{1}{\dot{x}}_{1}+k_{1}x_{1}=k_{c}\left(x_{2}-x_{1}\right)+f_{1}\\ m_{2}{\ddot{x}}_{2}+c_{2}{\dot{x}}_{2}+k_{2}x_{2}=k_{c}\left(x_{1}-x_{2}\right)+f_{2} \end{array}\right.$$

where $m_{i}$, $c_{i}$, $k_{i}$, $x_{i}$, $f_{i}$ (i=1, 2) represent the effective mass, effective damping, effective stiffness, vibration displacement, and external actuation force of resonators, respectively, and $k_{c}$ represents the coupling stiffness.

For the WCRs system, the effective mass, effective damping, and effective stiffness of the two resonators are assumed to be ideally symmetric, i.e., $m_{1}=m_{2}=m$, $c_{1}=c_{2}=c$, $k_{1}=k_{2}=k$. Due to the extremely high Q-factor of the resonator under vacuum packaging, the influence of damping terms is typically neglected in calculations. In the absence of any stiffness perturbation input, the expressions for the eigenvalues (resonant frequencies) and eigenstates (ARs) are derived as follows:

(2a) $$\left\{\begin{array}{l} \omega_{ip}^{2}\approx \omega_{0}^{2}\\ \omega_{oop}^{2}\approx \omega_{0}^{2}(1+2\kappa ) \end{array}\right.$$

(2b) $$\left\{\begin{array}{l} AR_{ip}=1\\ AR_{oop}=-1 \end{array}\right.$$

where $\omega_{0}^{2}=k/m$; $\kappa =k_{c}/k$ is the normalized coupling stiffness, $\omega_{ip}$, $\omega_{oop}$ are the resonant angular frequencies of IP mode and OOP mode, respectively, and $AR_{ip}$, $AR_{oop}$ are the ARs of the two modes, respectively. Figure 2 shows the mode shapes in three cases, namely without acceleration perturbation, with positive acceleration perturbation (Acc > 0), and with negative acceleration perturbation (Acc < 0).

This accelerometer adopts a differential input configuration. In this model, once the proof mass is affected by the inertial force, it synchronously alters the equivalent stiffness of both resonators. Particularly noteworthy is that, under ideal operating conditions, the stiffness perturbations experienced by these two resonators exhibit perfect symmetry: the perturbations are equal in magnitude, but their effects are diametrically opposed; that is $k_{1}=k-∆k,\;k_{2}=k+∆k$ (∆k means the stiffness perturbation due to acceleration). The expressions for the eigenvalues and eigenstates of both modes under the situation (Δk≠0) are as follows as follows:

(3a) $$\left\{\begin{array}{l} \omega_{1}^{2}\approx \frac{\left(k+k_{c}\right)}{m}-\sqrt{\frac{k_{c}^{2}+{\Delta k}^{2}}{m^{2}}}\\ \omega_{2}^{2}\approx \frac{\left(k+k_{c}\right)}{m}+\sqrt{\frac{k_{c}^{2}+{\Delta k}^{2}}{m^{2}}} \end{array}\right.$$

(3b) $$\left\{\begin{array}{l} AR_{1}=\frac{∆k+\sqrt{{∆k}^{2}+k_{c}^{2}}}{k_{c}}\\ AR_{2}=\frac{∆k-\sqrt{{∆k}^{2}+k_{c}^{2}}}{k_{c}} \end{array}\right.\;\;\;\;\;\;\;$$

When $k_{c}<0$ (for this device) and $\Delta k/k_{c}\gg 0$, the resonant frequencies and the amplitude ratios of both the in-phase mode and the out-of-phase mode can be reduced as follows:

(4a) $$\left\{\begin{array}{l} \omega_{oop}^{2}=\omega_{1}^{2}\approx \omega_{0}^{2}(1+\kappa +\varepsilon )\\ \omega_{ip}^{2}=\omega_{2}^{2}\approx \omega_{0}^{2}(1+\kappa -\varepsilon ) \end{array}\right.$$

(4b) $$\left\{\begin{array}{l} AR_{oop}=AR_{1}\approx -\frac{\kappa }{2\varepsilon }\\ AR_{ip}=AR_{2}\approx \frac{2\varepsilon }{\kappa } \end{array}\;\;\;\;\;\;\;\;\;\;\;\;\;\;\right.$$

where ε=Δk/k is the normalized stiffness perturbation.

According to the above equations, we observe that the resonant frequencies and ARs of the two modes vary with changes in stiffness perturbation.

### 2.3. Feedback-Control Voltage Output Sensitivity

**Figure 4:**
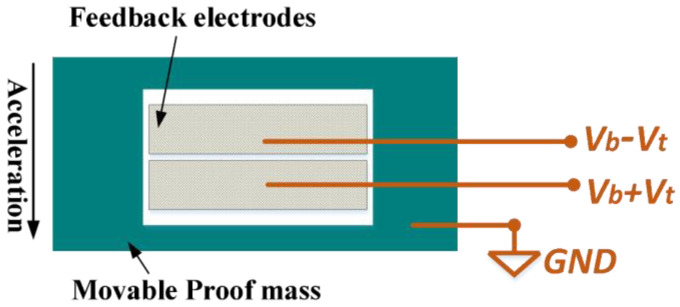
The feedback-control voltage ($V_{t}$) application method for the force-rebalance control scheme.

For the force rebalance control scheme of the ML-RXL, the system takes the feedback-control voltage as the output metric. The feedback electrodes contain two sets of electrodes that generate electrostatic force in opposite directions for the proof mass, balancing the inertial force in the positive and negative directions, respectively. The voltage is applied to the feedback electrodes, and the specific application method is shown in Figure 4. The proof mass is connected to the electrical ground, and two sets of feedback electrodes are applied with different DC voltages associated with the feedback-control voltage ($V_{t}$). The feedback-control voltage is a flexibly adjustable DC voltage. Under ideal conditions, two sets of feedback electrodes are perfectly symmetric. Therefore, based on the flat-plate capacitance model, the electrostatic force between the feedback electrodes and the proof mass can be expressed as follows:

(5) $$F_{e}=\frac{1}{2}\frac{C_{0}}{d}{\left(V_{b}+V_{t}\right)}^{2}-\frac{1}{2}\frac{C_{0}}{d}{\left(V_{b}-V_{t}\right)}^{2}$$

where d is the gap of between the feedback electrodes and the proof mass, $V_{b}$ is the constant bias voltage, $C_{0}=\varepsilon A/d$ is the initial capacitance value of between feedback electrodes and the proof mass, A is the area value of the parallel-plate capacitance, and ε is the vacuum permittivity.

The above formula can be simplified to $F_{e}=\frac{2C_{0}}{d}V_{b}V_{t}$. The inertial force sensed by the proof mass is derived as follows:

(6) $$F_{a}=Ma$$

where M is the effective mass value of the proof mass and a is the acceleration along the sensitive axis direction. When the system is in a state of equilibrium, the inertial force is in equilibrium with the electrostatic force. The feedback-control voltage output sensitivity can be calculated from $F_{a}=F_{e}$ as follows:

(7) $$a=\frac{2\varepsilon A}{Md^{2}}V_{b}V_{t}\propto V_{t}$$

Based on an analysis of Equation (7), it can be clearly observed that the sensitivity for the control method is directly proportional to the bias voltage ($V_{b}$), which means that the magnitude of the sensitivity can be directly controlled by adjusting the value of $V_{b}$. Moreover, the relationship between the sense acceleration and the feedback-control voltage is a simple linear relationship.

### 2.4. Systems Analysis of the Force Rebalance Control Scheme

**Figure 5:**
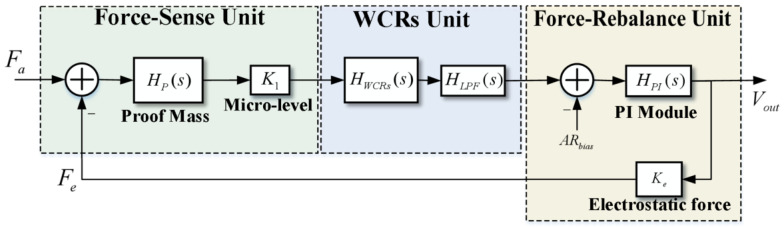
The loop diagrammatic sketch of the force rebalance control scheme.

The transfer function model of this control scheme is specifically analyzed. As shown in Figure 5, the proof mass module first detects the inertial force and the electrostatic force and transmits it to the micro-lever module; the force is then conducted into the stiffness perturbation of the WCRs, and the WCRs convert the stiffness perturbation into the AR output. In the force-rebalance unit, we use the error signal of the stable value of the AR output to the expected AR as the input of the PI module and output the voltage-control signal, which will generate electrostatic force on the proof mass, thus counteracting the inertial force.

The overall transfer-function model for the open loop of the system is described as follows:

(8) $$H_{open\text{-}loop}(s)=H_{p}(s)\cdot K_{1}\cdot H_{WCRs}(s)\cdot H_{LPF}(s)\cdot H_{PI}(s)$$

where $H_{p}(s)$, $H_{WCRs}(s)$, $H_{LPF}(s)$, and $H_{PI}(s)$ represent the transfer function of the second-order linear mass-spring-damper model for the proof mass, the WCRs, the low-pass filter (LPF), and the PI module, respectively, and $K_{1}$ represents the amplification factor of the micro-level.

Specifically, the transfer function of the WCRs remains unknown. Previous studies [24,45] have shown that there is a distinct response peak in the response curve of the transfer function. Therefore, the first assumption posits that the response model for the amplitude ratio output of the WCRs is characterized as a second-order linear resonant response model, which is described as follows:

(9) $$H_{WCRs}(s)=\frac{S_{AR}{\left(∆\omega \right)}^{2}}{s^{2}+\frac{∆\omega }{Q_{AR}}s+{\left(∆\omega \right)}^{2}}$$

where ∆ω is the angular frequency difference of two modes for the WCRs and $S_{AR}$ is the sensitivity factor of the AR output curve at different AR points. The model of the WCRs has been constructed in Simulink, and this initial assumption has been validated through simulation experiments (specific details of the simulation validation are presented in Section 3). The simulation results confirm that this assumption is applicable for small-signal dynamic acceleration inputs.

The response model of the proof mass can be approximated as a second-order linear resonance model. However, since the fundamental frequency of the proof mass (~2 kHz) is much larger than the frequency difference between the two modes of the WCRs, the response of the proof mass in the frequency range below the mode frequency difference can be approximated solely as the response to a DC signal input. This serves as the second assumption, which can be derived as follows:

(10) $$H_{p}(s)=\frac{1}{m_{p}s^{2}+c_{p}s+k_{p0}}\approx \frac{1}{k_{p0}}$$

where $m_{p},\;\;c_{p}$, $k_{p0}$ represent the effective mass, effective damping, and effective stiffness of the proof mass, respectively. The LPF adopts the first-order low-pass filter, and its transfer function is described as follows:

(11) $$H_{LPF}(s)=\frac{1}{1+\frac{s}{\omega_{LPF}}}$$

The PI module is taken as the basic first-order PI control mode. Its transfer function is described as follows:

(12) $$H_{PI}(s)=\left(K_{p}+\frac{K_{i}}{s}\right)$$

Therefore, the overall transfer function model for the closed loop of the system in the force rebalance control scheme is described as follows:

(13) $$H_{close\text{-}loop}(s)=\frac{H_{open\text{-}loop}(s)}{1+K_{e}H_{open\text{-}loop}(s)}=\frac{K_{1}\cdot S_{AR}{\left(∆\omega \right)}^{2}\cdot \left(K_{p}s+K_{i}\right)}{\left(k_{p0}\right)\cdot \left(s^{2}+\frac{∆\omega }{Q_{AR}}s+{\left(∆\omega \right)}^{2}\right)\cdot \left(1+\frac{s}{\omega_{LPF}}\right)\cdot s+K_{e}\cdot K_{1}\cdot S_{AR}{\left(∆\omega \right)}^{2}\cdot \left(K_{p}s+K_{i}\right)}$$

where $K_{e}$ is the ratio of the electrostatic force to the applied voltage. According to the Routh stability criterion, we compile the Routh calculation array table as follows:

(14) $$\begin{array}{l} \begin{array}{ccc} s^{4}: & a_{4} & \begin{array}{cc} a_{2} & a_{0} \end{array} \end{array}\\ \begin{array}{ccc} s^{3}: & a_{3} & a_{1} \end{array}\\ \begin{array}{l} \begin{array}{ccc} s^{2}: & b_{1} & b_{2} \end{array}\\ \begin{array}{cc} s^{1}: & c_{1} \end{array}\\ \begin{array}{cc} s^{0}: & d_{1} \end{array} \end{array} \end{array}$$

where

(15) $$\left\{\begin{array}{l} \begin{array}{l} a_{4}=k_{p0}\frac{1}{\omega_{LPF}}\\ a_{3}=k_{p0}+k_{p0}\frac{1}{\omega_{LPF}}\frac{∆\omega }{Q_{AR}}\\ a_{2}=k_{p0}\frac{∆\omega }{Q_{AR}}+k_{p0}\frac{1}{\omega_{LPF}}{\left(∆\omega \right)}^{2} \end{array}\\ \begin{array}{l} a_{1}=k_{p0}{\left(∆\omega \right)}^{2}+K_{e}\cdot K_{1}\cdot S_{AR}{\left(∆\omega \right)}^{2}\cdot K_{p}\\ a_{0}=K_{e}\cdot K_{1}\cdot S_{AR}{\left(∆\omega \right)}^{2}\cdot K_{i}\\ b_{1}=a_{2}-\frac{a_{1}a_{4}}{a_{3}} \end{array}\\ \begin{array}{l} b_{2}=a_{0}\\ c_{1}=a_{1}-\frac{a_{3}b_{2}}{b_{1}}\\ d_{1}=a_{0} \end{array} \end{array}\right.$$

When the coefficients in the first column of the Routh calculation array table are positive, the system is stable; that is, all the roots of the eigenequation are located in the left half plane of the root plane. We must meet the following conditions as follows:

(16) $$\left\{\begin{array}{c} \begin{array}{c} a_{4}>0\\ a_{3}>0\\ b_{1}>0 \end{array}\\ \begin{array}{c} c_{1}>0\\ d_{1}>0 \end{array} \end{array}\right.$$

The formula can eventually be reduced to the following:

(17) $$\left\{\begin{array}{c} a_{2}a_{3}-a_{1}a_{4}>0\\ a_{1}b_{1}-a_{3}a_{0}>0 \end{array}\right.$$

The parameters are selected according to the above stability criterion of the model for stability simulation verification, and the final control parameters are determined through the balance of response speed and stability accuracy.

## 3. Simulation Validation

**Figure 6:**
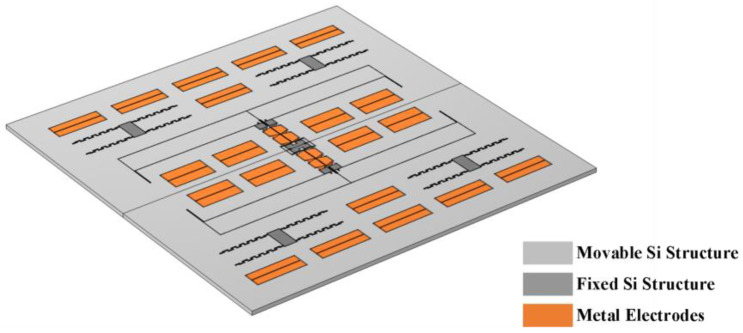
Perspective view of the device structure of the ML-RXL.

Considering the standard silicon on insulator (SOI) micro-fabrication process (the specific process steps are described in Section 4), the design of a novel ML-RXL that matches the previously proposed control scheme is presented. This specific structure is shown in Figure 6, where the light-gray part represents the movable silicon structure, the dark-gray part represents the fixed silicon structure, and the orange part symbolizes the fixed silicon electrode structure. The basic parameters of the device are summarized in Table 1.

In the design process, this work focuses on the precise control of the normalized coupling stiffness, with a design value set to about −3.2 × 10^−4^. To verify the effectiveness of the design, we implement a comprehensive simulation and analysis of the devices with this coupling stiffness using finite element analysis (FEA) software (COMSOL 5.6).

### 3.1. The FEA Results

**Figure 7:**
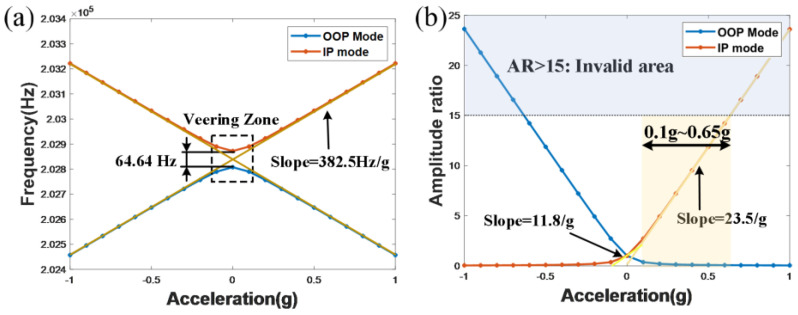
The output metrics characterization of both the IP mode and the OOP mode for the ML-RXL with $\kappa =-3.2\times 10^{-4}$, (**a**) the resonant frequencies, (**b**) the ARs.

The resonant frequency shift and the AR shift of both modes are simulated by using FEA software, which change in response to the acceleration input. The simulation results are shown in Figure 7, which demonstrates that the minimum frequency-split of two coupled modes is 64.64 Hz (corresponding to the normalized coupling stiffness of −3.2 × 10^−4^), the simulation AR sensitivity is 23.5/g in the linear zone, and there is an intrinsic nonlinear relationship between the acceleration perturbation with the output metrics in the veering zone, where the acceleration perturbation is close to zero.

In deeply exploring the performance of the AR output, owing to the inherent constraints of the SNR, the AR exhibits significant nonlinear properties when the system suffers from a large stiffness perturbation. This nonlinear problem leads to a deviation between the measurement results and the theoretical expectation in practical detection. In addition, the limitation of the linear detection range [0.1–0.65 g] does not start from the zero-input point 0 g, and this limitation poses a significant barrier to the widespread application of the mode-localization sensing mechanism. In conclusion, a further reduction in the coupling stiffness for the WCRs results in an increased sensitivity of the amplitude ratio output; however, it is accompanied by a corresponding compression of the dynamic range. Additionally, there are still other problems that may emerge. Specifically, when the coupling stiffness is excessively diminished, it can lead to mode aliasing among the coupled modes. This phenomenon occurs when the frequency of the higher-frequency mode for the WCRs overlaps with those of the lower-frequency mode, which can produce distorted signals or hinder the accurate discrimination of the various modes. To mitigate these issues, it is essential for the design to regulate the coupling strength, thereby ensuring the stability of the resonator across the intended frequency range.

### 3.2. Response Characteristics of the WCRs

Based on the exact simulation results obtained, a complete and comprehensive dynamic model of the ML-RXL is successfully constructed in Simulink to verify the control scheme of the device. The closed-loop excitation scheme for the WCRs is implemented in Simulink 2018a, which uses the single-resonator drive method to effectively stimulate the WCRs, and ensure that both resonators can stabilize the resonance in the working mode. The key of this scheme is the precise excitation control of the WCRs through phase-locked loop (PLL) technology.

**Figure 8:**
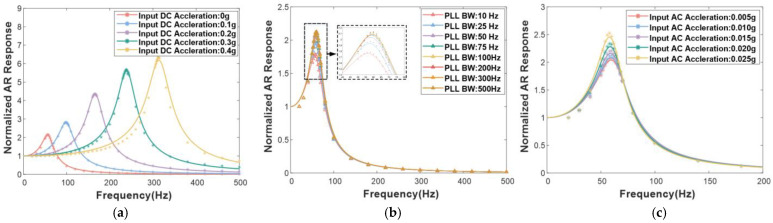
The simulation response results of the WCRs. (**a**) Varying frequency differences between the two modes when the PLL bandwidth is set at 500 Hz and the peak of the AC acceleration signal is 0.010 g; (**b**) Varying bandwidths of the PLL when the frequency difference of the two modes is set at 64.6 Hz and the peak of the AC acceleration signal is 0.010 g; (**c**) Varying peaks of the AC acceleration signal when the frequency difference of the two modes is set at 64.6 Hz and the PLL bandwidth is set at 500 Hz.

Based on the above setup, the response mechanism of the WCRs is studied. We investigate the mechanisms of how the different parameters influence the response properties through simulation experiments, and an empirical model of the response for the WCRs is finally established. The response behaviors of the WCRs under different conditions are simulated, as shown as Figure 8, such as varying frequency differences between the two modes (decided by the direct component of the acceleration signal), different bandwidths of the PLL, and different peaks of the acceleration signal. The response model is approximated in the second-order resonance response model, and the normalized response transfer function is described as follows:

(18) $$H_{WCRs}^{\prime }(s)=\frac{{\left(∆\omega \right)}^{2}}{s^{2}+\frac{∆\omega }{Q_{AR}}s+{\left(∆\omega \right)}^{2}}$$

It is observed from Figure 8a that the key parameter ∆ω is near the frequency difference between the two modes, and the $Q_{AR}$ value increases slowly with the increasing frequency difference. The curves mostly coincide, as shown in Figure 8b, which demonstrates that, when the bandwidth of the PLL is greater than the frequency difference, the $Q_{AR}$ value remains at the same value of about 2.4. It can be observed from Figure 8c that the $Q_{AR}$ value is mainly affected by the amplitude of the AC acceleration signal and, as the peak of the AC acceleration signal is higher, the $Q_{AR}$ value is also higher. In fact, the dynamic acceleration imposed in the simulation is relatively small.

### 3.3. Simulation of System Stability

**Figure 9:**
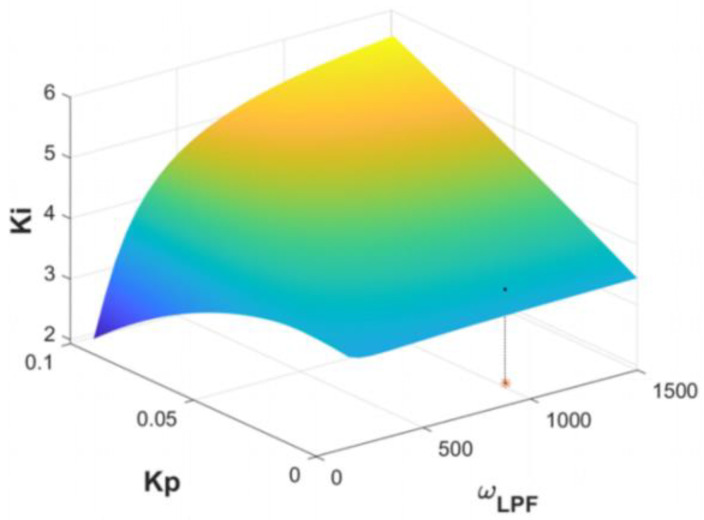
The limit surface of the system where the variable parameters are $\omega_{LPF}$,${\;K}_{p}$, and $K_{i}$. The * marked the parameters for a set of stable solutions of the system, specifically $\omega_{LPF}=2\pi \times$1000 rad/s,${\;K}_{p}=0.01$, $K_{i}=2$.

Based on the above analysis, the specific parameters of each module in the system loop are determined, as shown in Table 2. The parameters in the table are under the conditions that the amplitude ratio is near 1, and $\omega_{LPF}$, $K_{p}$, $K_{i}$ are selected as the variable parameters. The stable regions of the force rebalance control system are shown in Figure 9. The stable points determined by three parameters are under the limit surface. ${\;K}_{p}$, $K_{i}$ are the key parameters that influence the response speed and system stability. When the two parameters ($K_{p}$, $K_{i}$) increase, the system will quickly return to a steady state.

### 3.4. Voltage Output Characteristics for Force Rebalance Control Scheme

**Figure 10:**
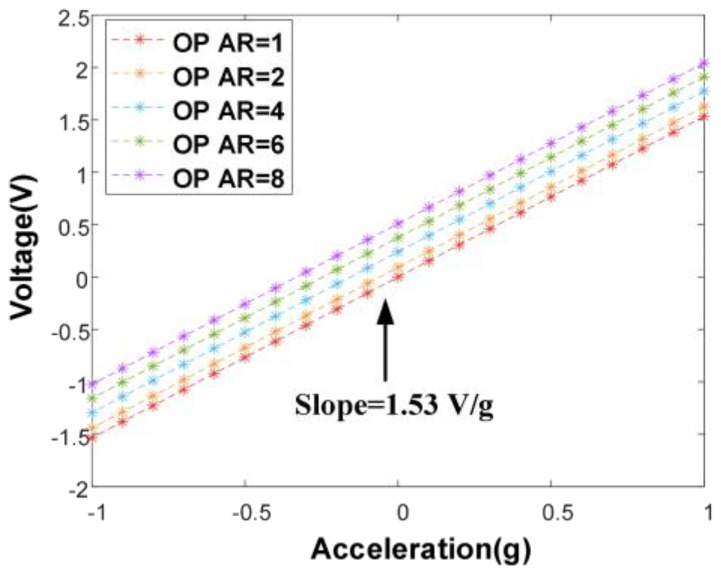
Voltage output characteristics of different OPs.

The Simulink model is constructed in the force rebalance control scheme on the basis of the closed-loop excitation scheme. The parameters are determined according to the previously established overall transfer model, and the characteristics of the feedback-control voltage output at different AR operation points in the whole range are then analyzed. A suitable set of parameters is selected ($\omega_{LPF}=2\pi \times$1000 rad/s,${\;K}_{p}=0.01$, $K_{i}=2$). It can be observed from Figure 10 that there is a constant sensitivity for different AR states, but that the detection acceleration range is slightly offset. In conclusion, the feedback-control voltage output shows a high linearity and has a broadened measurement range compared with the AR output.

## 4. Device Fabrication and Experimental Setup

### 4.1. Device Fabrication

**Figure 11:**
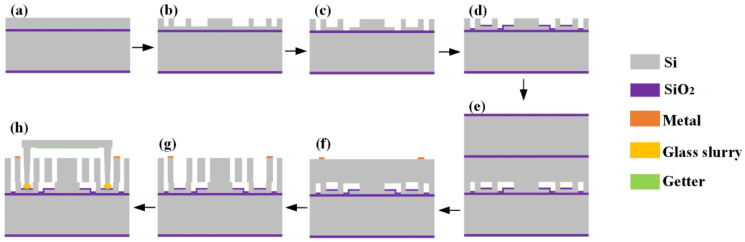
Fabrication process flow of the ML-RXL. (**a**) Prepare an SOI wafer; (**b**) etch shallow cavity; (**c**) pattern the bottom electrode; (**d**) deposite the silicon oxide in the shallow cavity; (**e**) prepare the second SOI wafer and bond two wafers together by the silicon-to-silicon bonding process; (**f**) remove the substrate layer and the oxygen layer, deposite and pattern the metal layer; (**g**) etch the device layer structure; (**h**) package in a vacuum environment by the glass slurry bonding process.

The ML-RXL is fabricated using the silicon micro-manufacture technology, and the core of the technology is the standard silicon on insulator (SOI) micro-fabrication process and the multilayer silicon wafer bonding process. The production process for this chip involves three pieces of wafers: two SOI wafers and a standard silicon wafer. Specifically, the first SOI wafer serves primarily as the substrate structure, and the carefully crafted shallow cavity structure on its device layer is key to achieving the release of the ML-RXL’s structure. The second SOI wafer’s device layer is used to build the core functional components of the ML-RXL, covering structures such as the proof mass, the WCRs, the micro-levers, and the electrodes. The third SOI wafer is used to realize the final package process, ensuring the integrity and functionality of the entire chip structure. A flowchart for the device manufacturing process reference is shown in Figure 11. The specific process is as follows: (a) the fabrication process starts with an SOI wafer (device layer thickness of 20 μm, buried oxygen layer thickness of 1 μm, and substrate layer thickness of 380 μm); (b) the shallow cavity in the device-layer of the SOI wafer is etched and formed by the DRIE process; (c) etch the silicon structure down to the buried oxygen layer, thereby completing the patterning of the bottom electrode; (d) the silicon oxide is deposited in the shallow cavity; (e) the second SOI wafer (device layer thickness of 50 μm, buried oxygen layer thickness of 1 μm, and substrate layer thickness of 380 μm) is prepared and cleaned, and its surface is polished for the next bonding process; the two SOI wafers are bonded together by the silicon-to-silicon bonding process; (f) the substrate layer and the oxygen layer of the second SOI wafer are removed by the etching process, and the metal layer is then deposited and patterned on the silicon surface; (g) the device layer structure is patterned via the DRIE process; finally, (h) the third standard silicon wafer is etched with deep cavity structures and the Ti-based absorber material is sputtered at the bottom of this structure; The third wafer is used as a cap, which is tightly bonded to the pre-treated wafer in a vacuum environment by the glass slurry bonding process, thereby achieving the package of the chip. During the bonding process, the equipment is able to reach ultimate vacuum conditions of 1 × 10^−5^ mbar and sintering temperatures up to 450 °C. The wafer is divided into die pieces after a laser-cutting process, which is prepared for the following experimental test. By testing the Q-value of resonators on the packaged chip, it can be predicted that the internal vacuum of the chip package is approximately within a range of 0.1 to 1 Pa.

### 4.2. Experimental Setup

**Figure 12:**
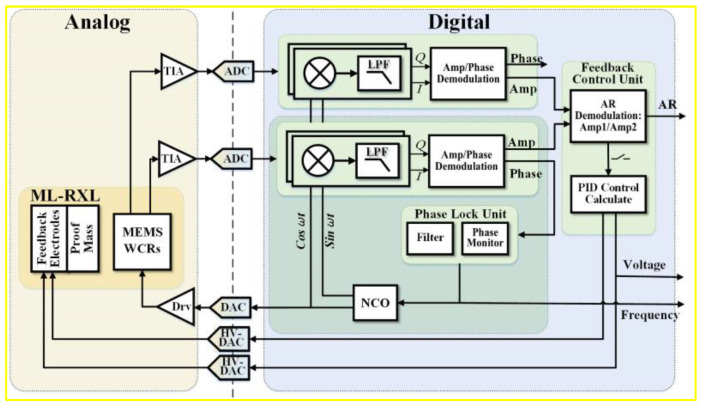
Schematic diagram of the force-rebalance control scheme for the ML-RXL.

The schematic diagram of the specific control principle for the ML-RXL is shown in Figure 12. Both control schemes are implemented using a digital-circuit method. At the digital circuit, the closed-loop excitation scheme is implemented through a PLL method, where the excitation signal is applied to the driving electrodes of the WCRs via a digital-to-analog converter (DAC). The force rebalance control scheme takes the AR output as the input of the force rebalance feedback loop and compares it with the set constant value of the AR. Then, the difference between the AR output and the set constant value is used to generate a control signal through a PID module, which is then applied to the feedback electrodes via a high-voltage digital-to-analog converter (HV-DAC).

**Figure 13:**
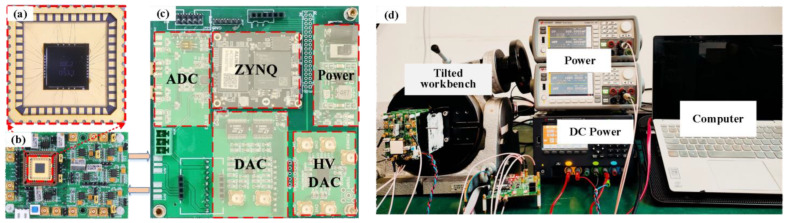
(**a**) The optical micro-graph of the packaged ML-RXL and the details of bonding wires. (**b**) Analog front-end signal processing circuit board. (**c**) Digital control system circuit board. (**d**) The experimental setup.

The accelerometer chip is glued to the middle of a standard 44-pin DIP ceramic chip carrier, and the signals of the accelerator chip are connected with the corresponding electrode pins of the chip carrier, as shown in Figure 13a. Therefore, we can monitor the movement currents caused by the vibration of the resonators via detecting the sense electrodes and effectively convert and amplify the weak current signals into voltage signals through an analog front-end signal-processing circuit, as shown in Figure 13b. The voltage signals from both resonators are collected by the analog-to-digital converter (ADC) and are transmitted to the digital control system for processing, as shown in Figure 13c. To test the output sensitivity of the ML-RXL, the device is fixed on a tilted workbench and is applied with an acceleration signal with a range of ±1 g by the gravity field through the change of rotation angle, as shown in Figure 13d.

## 5. Experimental Results and Discussion

### 5.1. AR Output Results

**Figure 14:**
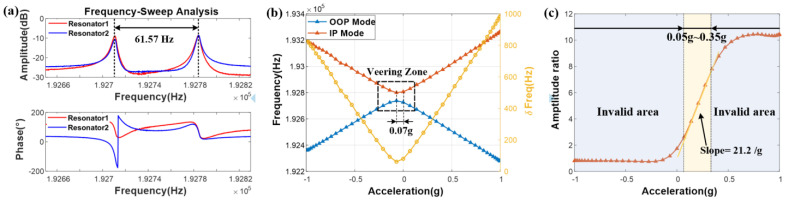
(**a**) The amplitude-frequency response and the phase-frequency response of both the modes for the device without perturbation. (**b**) The frequency shifts of both the modes tested in the open-loop measurement. (**c**) The AR shift of the IP mode tested in the measurement for the closed-loop excitation scheme.

First, the open-loop characterization of the device is tested by a frequency-sweep operation. In the open-loop experiment setup, the WCRs and the proof mass are maintained at 0 V, and the drive electrodes and the sense electrodes are given a DC voltage of 10 V, while an AC signal (5 mVpk) is superimposed on the drive ports to excite the resonators. As shown in Figure 14a, the resonance frequencies of both modes for the device without perturbation are 192,722.85 Hz and 192,784.42 Hz, respectively. Moreover, judging from the phase-frequency response, the IP mode has a higher frequency, and the mode with a lower frequency is the OOP mode, which is consistent with the theoretical analysis of the situation with κ<0. The frequency split is ~61.57 Hz (equal to the normalized coupling stiffness of −3.19 × 10^−4^).

In the open-loop measurement, the frequency shifts of both the IP mode and the OOP mode with a measurement range of ±1 g are tested, as exhibited in Figure 14b. The nonlinear veering zone is present in the situation, with an acceleration perturbation close to zero. As the perturbation increases, the frequencies of both modes change dramatically because the device adopts a differential input configuration, which is consistent with the phenomenon of the finite element simulations. The normalized frequency-shift sensitivity of the device is about 2364 ppm/g, while the sensitivity of the finite element simulations is 1885 ppm/g. Furthermore, compared with the finite element simulation results, as shown in Figure 7a, it can be observed that the acceleration input value corresponding to the smallest mode frequency difference is shifted. This is mainly caused by the following reasons: asymmetry of the device structure due to process errors, non-uniformity of the residual stress distribution due to process fabrication and bond fixation, and angular deviation of the actual sensitive axis of the ML-RXL from the axis to be measured. It is worth noting that the offset value has been significantly optimized to 0.07 g, which means that the WCRs can be directly operated in the state of the coupling without additional stiffness adjustment.

In addition, the AR shift of the IP mode is tested by the closed-loop excitation scheme. As indicated in Figure 14c, the results show that there is an obvious nonlinearity phenomenon among the measurement spans of the smaller and larger acceleration perturbations, which depend on the intrinsic nonlinear relationship and the SNR, respectively. Moreover, the experimental value of AR sensitivity is 21.2/g in the measurement range [0.05 g, 0.35 g]. Furthermore, it can be observed that, in addition to the two nonlinearities that cause the linear region of the AR output to decrease, there is a slight shift in the linear region of the AR compared to the finite element simulation results. The reason for this shift is the same as that of the shift of the corresponding point when the mode frequency difference reaches the minimum value.

### 5.2. Voltage Output Results

Owing to fabrication-process defects, there is usually a certain difference in the capacitance value of the feedback electrodes in actual devices. Therefore, it needs to quantify this difference by a pre-experimental calibration method. First, the efficiencies of different electrodes generating electrostatic force to balance the actual inertial acceleration by the force rebalance control scheme are examined. The voltage configuration follows the previous settings. In addition, the voltage generated by the single-electrode control method, rather than the double-electrode control method, as shown in Figure 4, is applied to two sets of feedback electrodes.

**Figure 15:**
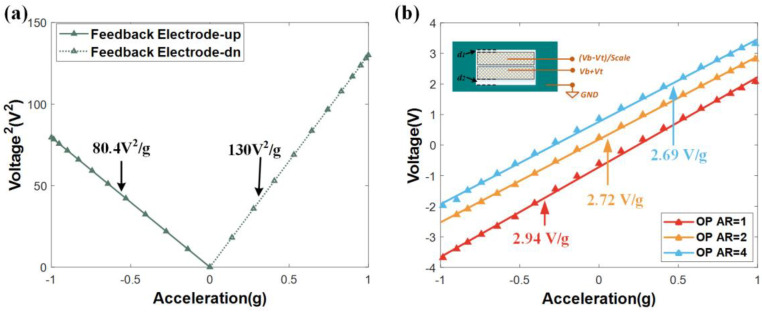
(**a**) The feedback-control voltage-output characteristics of the device for the single-electrode control method. (**b**) The feedback-control voltage output characteristics of different AR setups for the double-electrode control method of the force rebalance control scheme.

In the above configuration, the voltage output characteristic of the device during a ±1 g measurement range is measured when the operation point AR is 1, as exhibited in Figure 15a, which includes results for both sides of the feedback electrodes. From the results, it can be observed that the efficiencies of two sets of symmetric feedback electrodes differ significantly.

When implementing the force rebalance control scheme, we adjust the applied voltage on one side of the feedback electrodes by a corrective parameter to compensate for the asymmetry issue. Based on this approach, the feedback-control voltage-output characteristics of the different AR setups are tested, as shown in Figure 15b, which show that the sensitivity of the feedback-control voltage output is 2.94 V/g at the AR of 1. It can be observed that slight differences arise in the sensitivity for different AR states, which is because the electrostatic force on the proof mass can not only stabilize the WCRs at different AR states, but alter the capacitance on both sides of the feedback electrodes. Nonetheless, the feedback-control voltage output shows a highly linear range in the ±1 g range, and its ideal linear range completely exceeds ±1 g in theory, which is only affected by the voltage-output range.

### 5.3. Stability Results

**Figure 16:**
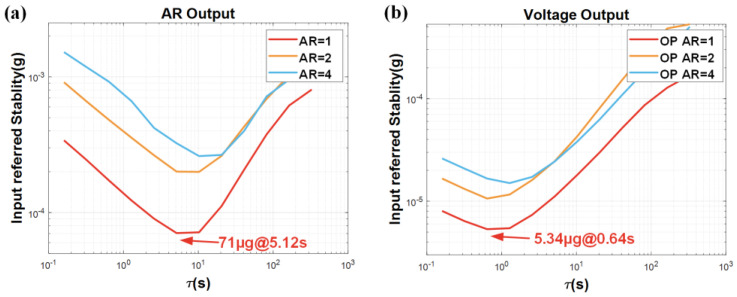
The Allan variance results of the ML-RXL for (**a**) a closed-loop excitation scheme and (**b**) a force-rebalance control scheme.

To evaluate the stability characteristic of the device, this sensor employs the closed-loop configurations, specifically including the closed-loop excitation scheme and the force rebalance control scheme. In the experiments, we continuously collect the AR and feedback-control voltage data for 10 min at a sampling rate of 100 Hz and analyze the stability of the two schemes by calculating the Allan deviations from the different output indices. Figure 16a visually shows the bias instability (BI) of the AR output for the closed-loop excitation scheme, while Figure 16b reveals the BI of the feedback-control voltage output in the force rebalance control scheme.

This sensor can achieve a BI of 71 μg at 5.12 s in a conventional closed-loop excitation scheme, while a better BI of 5.34 μg at 0.64 s is achieved in the force rebalance control scheme. By comparing the measurement results at different AR values, it can be observed that there is a decreasing trend of the BI results as the AR increases. Specifically, when adopting the force rebalance control scheme, the feedback-control voltage output achieves a best BI for the AR of 1; and the stability results are worse for the AR of 2 and 4, which are 10.6 μg and 15.0 μg, respectively.

When the amplitude ratio is used as the output signal, the dynamic range of the ML-RXL is constrained by the measurement range and resolution of the amplitude ratio output, resulting in a limited dynamic range of only 72.5 dB. In fact, there is still a performance gap in the test results obtained from the current testing system when compared to those acquired using a lock-in amplifier for conventional closed-loop excitation. This indicates that there is potential for further optimization of the system to enhance its performance.

When the force rebalance control strategy is employed with feedback-control voltage as the output, the tested dynamic range of the ML-RXL is 111 dB. The linear range only depends on the voltage-output range, which exceeds the ±1 g acceleration range applied during the experiment. Therefore, the actual dynamic range of the device is greater than the tested value.

### 5.4. Noise Floor Results

**Figure 17:**
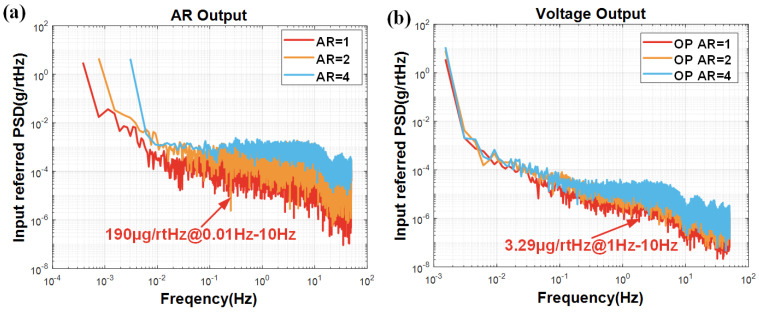
The PSD analysis results of the ML-RXL for (**a**) a closed-loop excitation scheme and (**b**) a force rebalance control scheme.

To further investigate the properties of the two strategies, the input referred power spectral-density (PSD) results for the two schemes are shown in Figure 17. The ML-RXL under the force rebalance control shows a significant advantage in noise performance, and its noise floor is only 3.29 μg/rtHz at AR = 1, which is decreased by an order of magnitude compared with the 190 μg/rtHz in the closed-loop excitation scheme.

The different curves presented in Figure 17 represent the measurement results at different AR states. It is found that, when the AR tends to 1, the noise performance shows a better trend. This result basically agrees with previous studies [12,46], and it can be obtained at the optimum noise floor for the AR output at an AR of 1.23. Specifically, when the AR is set to 4, the noise floor is 11.1 μg/rtHz; when the AR is controlled at 2, the noise floor is reduced to 6.62 μg/rtHz; when the AR is accurately controlled at 1, the noise floor will get lower.

## 6. Conclusions

In this paper, a force rebalance control scheme for the ML-RXL has been first proposed. According to the control scheme, an ML-RXL has been designed and manufactured. In addition, we have built a response model of the WCRs, as well as a simplified model of the force rebalance control system, and analyzed its properties through simulations. It is proved that the force rebalance control scheme can improve the sense range, which opens up a broader prospect for the application of the ML-RXL.

Moreover, the control circuit system by analog and digital methods is realized. When the sensor works in the traditional closed-loop excitation scheme, the AR sensitivity is 21.2/g, and the effective linear range is only [0.05 g, 0.35 g]. However, the feedback voltage output in the force rebalance control scheme proposed above, which can maintain a highly linear output within the measurement range of ±1 g, has a sensitivity of 2.94 V/g (AR = 1). The measurement range is expanded by ~6.7 times. This method can realize the decoupling of the sensitivity and measurement range of the WCRs, and it can broaden the effective sense range while maintaining the ultra-high sensitivity detection. In the full range, the disturbance signal of the WCRs is extremely small, so the theoretical nonlinearity of the coupled resonators can be ignored.

The noise floor of the AR output for the traditional scheme is 190 μg/rtHz, and the optimal BI result is 71 μg at 5.12 s. By the force rebalance control scheme, the noise floor is optimized to 3.29 μg/rtHz, and the BI is further improved to 5.34 μg at 0.64 s. We are studying in more detail how to optimize the noise floor of the system and improve the BI of the ML-RXL. This ongoing work involves the optimization of system parameters, the selection of feedback loop input signals, and the selection of working points.

## Figures and Tables

**Table 1:** Basic Design Parameter of the ML-RXL.

Parameter	Dimensions
Total chip size	6.65 mm × 6.65 mm
Device layer thickness	50 μm
Proof mass	1.87 mg
Resonant frequency of proof mass	2156 Hz
Feedback electrode capacitance	1.59 pF
Feedback electrode gap	2 um
Beam size for resonators	400 μm × 6 μm
Beam resonant frequency	~202.9 kHz
Normalized coupling stiffness	−3.2 × 10^−4^

**Table 2:** Basic Parameters of the Force Rebalance Loop.

Symbol	Parameter	Value
$m_{p}$	Effective proof mass	1.87 mg
$c_{p}$	Effective damping of proof mass model	$4.54\times 10^{-6}\;N\cdot s/m$
$k_{p0}$	Effective stiffness of proof mass model	275 N/m
$K_{1}$	Amplification factor of the micro-level	$3.99\times 10^{7}\;N/m^{2}$
$S_{AR}$	Sensitivity of WCRs for stiffness perturbation at AR = 1	$43.5/\left(N/m\right)$
∆ω	Peak frequency of WCRs response model at AR = 1	2π×64.6 rad/s
$Q_{AR}$	Peak quality factor of WCRs response model at AR = 1	~2.40
${SF}_{AR}$	Sensitivity of ML-RXL for acceleration perturbation at AR = 1	11.8/g
$K_{e}$	Sensitivity of electrostatic force to voltage	$1.195\times 10^{-5}$ N/V
${SF}_{V}$	Sensitivity of virtual electrostatic acceleration to voltage	1.53 V/g

## Data Availability

The original contributions presented in this study are included in the article. Further inquiries can be directed to the corresponding author.
